# MicroRNA 10b promotes abnormal expression of the proto-oncogene c-Jun in metastatic breast cancer cells

**DOI:** 10.18632/oncotarget.11000

**Published:** 2016-08-02

**Authors:** Revital Knirsh, Iris Ben-Dror, Shira Modai, Noam Shomron, Lily Vardimon

**Affiliations:** ^1^ Department of Biochemistry and Molecular Biology, George S. Wise Faculty of Life Sciences, Tel Aviv University, Tel Aviv, Israel; ^2^ Department of Cell and Developmental Biology, Sackler Faculty of Medicine, Tel Aviv University, Tel Aviv, Israel

**Keywords:** miR10-b, c-Jun, NF1, RhoC, metastatic breast cancer

## Abstract

MicroRNAs have been shown to act as oncogenes or tumor suppressers via various cellular pathways. Specifically, in breast cancer, upregulation of miR-10b is positively associated with aggressiveness of tumors. However, the mechanism by which miR-10b contributes to cell malignancy is largely unknown. Here we show that at the receiving end of the miR-10b pathway is the proto-oncogene c-Jun, a transcription factor that plays a critical role in stimulation of cell proliferation and tumor progression. c-Jun is known to be translationally activated by loss of cell contacts or restructuring of the cytoskeleton. A comprehensive analysis of miRNA expression exhibited a significant increase in miR-10b expression. This was supported by analysis of breast cancer cells, which showed that loss of E-cadherin in metastatic cells is accompanied by elevation of miR-10b and interestingly, by a marked increase in accumulation of c-Jun. Silencing miR-10b in metastatic breast cancer cells leads to a decline in c-Jun expression, whereas overexpression of miR-10b in HaCaT cells is sufficient to elevate the accumulation of c-Jun. The increase in c-Jun protein accumulation in metastatic cells is not accompanied by an increase in c-Jun mRNA and is not dependent on MAPK activity. Knockdown and overexpression experiments revealed that the increase is mediated by NF1 and RhoC, downstream targets of miR-10b that affect cytoskeletal dynamics through the ROCK pathway. Overall, we show the ability of miR-10b to activate the expression of c-Jun through RhoC and NF1, which represents a novel pathway for promoting migration and invasion of human cancer cells.

## INTRODUCTION

The transcription factor c-Jun is a protooncogene that plays a key role in cell proliferation and tumor progression. It belongs to the AP-1 transcription factor family, known to upregulate the expression of genes involved in the cell cycle machinery, downregulate the expression of tumor suppressors and contribute to the migration and invasion processes through induction of genes such as matrix metalloproteinases (MMPs) [1, 2], Conditional inactivation of the c-Jun gene, or inhibition of c-Jun/AP1 activity by the addition of dominant-negative c-Jun (TAM67), or siRNA, inhibit cell proliferation and suppress the invasive ability of tumor cells [3, 4]. Conversely, overexpression of c-Jun results in malignant transformation of various cell lines [1, 5]. Although it is clear that c-Jun can function as an oncogene, the molecular mechanism that upregulates c-Jun levels in cancer cells is only partially understood.

Exposure of cells to growth factors or various extracellular stressors, including, ultraviolet irradiation and genotoxic stress, may provide the trigger to upregulate c-Jun expression. In the case of such external signals, the control of c-Jun expression is mainly at the transcriptional level and the mechanism is dependent on MAP kinase (MAPK) signaling pathway activity [6]. There have been recent reports that the expression of c-Jun can also be regulated by intracellular signals involving cell-cell contact or cytoskeletal components [7–11]. Accumulation of c-Jun is markedly elevated upon cell dispersion or inhibition of the activity of the adhesion molecule E-cadherin [7]. Other inducers include depolymerization of the cytoskeleton by an overexpression of cofilin [12] or addition of cytoskeleton disrupting agents [7, 9, 10]. However, in contrast to the external signals that upregulate the transcription of the c-Jun gene, loss of E-cadherin or restructuring of the cytoskeleton exert their control at the level of translation of the c-Jun transcript [7, 10].

MicroRNAs (miRNAs) and short non coding RNAs dominantly regulate gene expression by binding to the 3′UTR of the mRNA. Misregulation of miRNAs has been associated with various diseases, in particular cancer. Specifically, miR-10 was shown to promote migration and invasion in human breast cancer cells [13]. In these cells, as in most tumors of epithelial origin, the molecular program driving the metastasis is the epithelial mesenchymal transition (EMT), a fundamental process in the progression of tumors toward the invasive phase. This process is characterized by loss of function of the adhesion molecule E-cadherin and by extensive cytoskeleton rearrangement [14–16]. Upregulation of E-cadherin in tumor cells reverses the invasive phenotype to a benign one [14, 17–19]. Given the physiological role of c-Jun in tumor promotion, loss of E-cadherin might contribute to tumor malignancy through upregulation of c-Jun. Here we show that loss of E-cadherin in human metastatic breast cancer cells is indeed accompanied by increased concentrations of c-Jun protein and a concomitant increase in cell malignancy. Most interestingly, the elevation of c-Jun is not mediated by MAPK, but occurs via a novel mechanism that is activated by miR-10b and mediated by RhoC and NF1.

## RESULTS

### Expression of c-Jun is controlled by miR-10b

**Figure 1:**
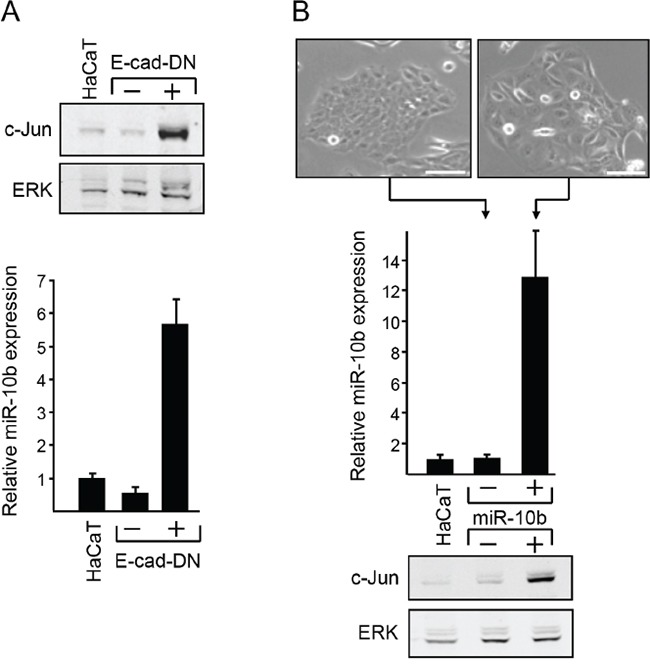
Functional link between E-cadherin, c-Jun and miR10b **A.** RNA samples from stably transfected HaCaT cells that expresses (+) or do not express (−) dominant negative E-cadherin (E-cad-DN) or from the parental HaCaT cells, were analyzed by quantitative RT-PCR using primers for miR-10b and U6 snRNA. Parallel protein samples were assayed by western blotting to determine protein level of c-Jun and ERK. **B.** HaCaT cells were stably transfected with a miR-10b expression vector (+) or an empty vector (−) as control. Representative phase-contrast images of the cells are shown (scale bar: 100μm). RNA samples from untransfected or transfected cells were analyzed by Real Time RT-PCR using primers for miR-10b and U6 snRNA. Parallel protein samples were assayed by western blotting to determine the protein level of c-Jun and ERK.

Loss of cell-cell contacts upregulates the translation of the c-Jun transcript [7]. Considering the emerging role of MicroRNAs (miRNAs) in cancer development, we examined the possible involvement of miRNAs in this process. We used human keratinocyte HaCaT cells that were stably transfected with dominant negative E-cadherin (E-cad DN) [7]. In these cells, loss of E-cadherin induces the translation of c-Jun, resulting in a considerable increase in c-Jun protein concentration (Figure 1A, upper panel). Global analysis of miRNA expression in control and E-cad DN cells revealed that miR-10b was the most significantly altered miRNA. Quantitative RT-PCR analysis showed that the level of miR-10b in E-cad DN cells was almost 6-fold higher than that in control cells (Figure 1A, lower panel). To assess the possible involvement of miR-10b in c-Jun regulation, we stably transfected HaCaT cells with an expression vector containing miR-10b or control. Expression of miR-10b was monitored by quantitative RT-PCR (Figure 1B, middle panel). Analysis of the transfected cells revealed that ectopic expression of miR-10b resulted in altered epithelial morphology (Figure 1B, upper panel) and caused a significant augmentation of the level of c-Jun protein (Figure 1B, lower panel). This observation suggests that E-cadherin control of c-Jun expression is mediated, at least in part, by miR-10b.

### miR-10b upregulates c-Jun expression *via* RhoC and NF1

**Figure 2:**
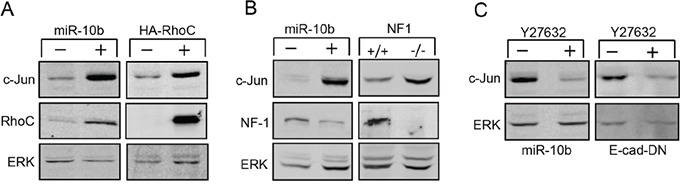
Upregulation of c-Jun is mediated by RhoC and NF-1 **A.** Protein analysis of c-Jun, RhoC and ERK in HaCaT cells stably transfected with miR10b (+) or control (−) construct (left panel) or with the constitutive active HA- RhoC (+) or control (−) construct (right panel). **B.** Protein analysis of c-Jun, NF1 and ERK in HaCaT cells stably transfected with miR10b (+) or control (−) construct (left panel) or in wild type (NF1+/+) or NF1 knockout (NF1−/−) MEFs (right panel). **C.** Protein analysis of c-Jun and ERK in HaCaT cells stably transfected with miR10b (left panel) or with E-cad-DN (right panel) that were cultured with (+) or without (−) the ROCK inhibitor, Y27632. The experiments were repeated at least three times and representative immunoblots are shown.

Each miRNA has the potential to bind a large set of mRNAs. The ‘targeting’ of mRNAs is identified by using computational prediction tools. However, several of these tools failed to identify potential miR-10b target sites in the c-Jun transcript. Such sites have been previously identified in the mRNA of the homeobox D10 (HOXD10) [13] and neurofibromin 1 (NF1) [20], two proteins that are implicated in cytoskeletal dynamics. HOXD10 is a transcriptional repressor of RhoC. Inhibition of HOXD10 by miR-10b results in increased expression of RhoC [13, 21], which activates a signaling pathway that alters cytoskeletal organization. This pathway is negatively regulated by NF1, which blocks the activity of RhoC downstream effectors [22, 23]. Considering that cytoskeletal dynamics has a critical role in activation of c-Jun translation [7, 10], we examined whether miR-10b enhances the expression of c-Jun via this pathway. We first examined whether transfection of miR-10b into HaCaT cells causes an increase in expression of RhoC. Western blot analysis indeed showed that overexpression of miR-10b resulted in a 6-fold increase in RhoC expression (Figure 2A, left panel). Overexpression of constitutively active (G14V) RhoC (HA-RhoC) elevated the levels of c-Jun considerably (Figure 2A, right panel) indicating a role for RhoC in c-Jun regulation. As expected, overexpression of miR-10b also repressed the expression of NF1. Accumulation of NF1 in miR-10b transfected cells was 9-fold lower than that in control cells (Figure 2B, left panel). To assess the involvement of NF1 in c-Jun regulation we used NF1 knockout (NF1^−/−^) mouse embryonic fibroblasts (MEF) and congenic WT (NF1^+/+^) cells as control [24]. The levels of c-Jun were found to be considerably elevated in the NF1 knockout fibroblasts (Figure 2B, right panel). The effect of RhoC and NF1 on cytoskeletal dynamics is known to be mediated by downstream effectors, the most important of which is the Rho-associated coiled-coil forming kinase, ROCK [25, 26]. We examined whether treatment with the ROCK specific inhibitor, Y27632, could affect the expression of c-Jun. When miR-10b transfected cells were assayed for c-Jun expression in the presence or absence of Y27632, treatment with the inhibitor resulted in a marked reduction in the amount of c-Jun protein (Figure 2C, left panel). Similarly, addition of Y27632 to E-cad DN cells, also down regulated the expression of c-Jun (Figure 2C, right panel). These findings implicate the functional association of RhoC and NF1 in the control of c-Jun expression and suggest that they are responsible for the miR-10b-mediated upregulation of c-Jun, following the loss of E-cadherin.

### Posttranscriptional activation of c-Jun expression in human breast cancer cells

**Figure 3:**
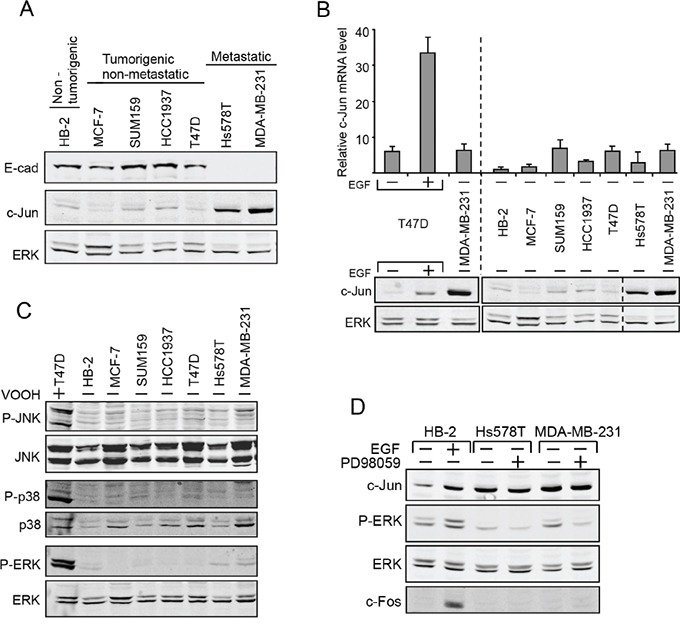
Upregulation of c-Jun in human metastatic breast cancer cells in post-transcriptionally controlled Breast epithelial cell line (HB-2), breast cancer cell lines that are not metastatic (MCF-7, SUM159, HCC193, T47D) or metastatic breast cancer cell lines (Hs578T, MDA-MB-23) were analyzed. **A.** Protein level of E-cad, c-Jun and ERK was determined by western blotting. **B.** RNA samples were assayed by quantitative RT-PCR using primers for c-Jun and GAPDH. T47D cells untreated or treated with EGF were used as a control. Parallel protein samples were assayed by western blotting to determine the level of c-Jun and ERK. **C.** Protein samples were assayed by western blotting using anti total or phosphorylated (P) JNK, ERK or p38 antibodies. T47D cells treated with VOOH (+) were used as a control. **D.** Western blot analysis of c-Jun, c-Fos, ERK and phospho-ERK (P-ERK) expression in cells treated (+) or untreated (−) with EGF or with the MEK inhibitor PD98059.

Loss of E-cadherin in most cancers of epithelial origin occurs concomitantly with progression towards tumor malignancy. To examine whether this loss of E-cadherin is associated with increased levels of c-Jun protein, we compared a non-tumorigenic human breast epithelial cell line (HB-2) to tumorigenic breast cancer cell lines that either are metastatic (Hs578T and MDA-MB-231) or non-metastatic (MCF-7, SUM159, HCC1937 and T47D). Western blot analysis showed that the level of expression of E-cadherin is inversely correlated with the expression of c-Jun: E-cadherin is high in non-tumorigenic or non-metastatic cells, but low in the metastatic lines, while expression of c-Jun is high in the metastatic cells and low in the others (Figure 3A).

The increase in c-Jun expression that is dependent on cell-contact is post-transcriptionally controlled [7]. The possibility that the reduction in E-cadherin seen in metastatic breast cancer cells is functionally related to the upregulation of c-Jun implies that there too, the increase is not transcriptionally but rather post-transcriptionally controlled. In support of this hypothesis, quantitative RT-PCR analysis revealed that in contrast to the increase of c-Jun mRNA seen after treatment with EGF, which is known to elevate levels of c-Jun protein by working at the level of transcription, the increase in c-Jun protein seen in metastatic breast cancer cells was not accompanied by upregulation of c-Jun mRNA (Figure 3B). Control of both c-Jun transcription and protein stability is known to be mediated mainly by the MAPK pathways, particularly by JNK, ERK and p38. We used antibodies against phosphorylated JNK, ERK and p38 to examine the extent of activation of these MAPK pathways in breast cancer cells using the general phosphatase inhibitor, peroxovanadate (VOOH), as control. The results showed that the levels of phospho-JNK (P-JNK), phospho-p38 (P-p38), and phospho-ERK (P-ERK) were equally low in all the breast cell lines used, indicating that the MAPK pathway is not particularly activated in metastatic breast cancer cells (Figure 3C). In some experiments there was some increase in ERK phosphorylation, but addition of PD98059, a specific mitogen-activated protein kinase kinase (MEK) inhibitor to inhibit the ERK pathway, did not impede the accumulation of c-Jun (Figure 3D). In accordance with these findings, the expression of c-Fos, a transcription factor that is upregulated by MAPK activity, was barely detectable, in both normal and metastatic breast cancer cells. This is in contrast to the increase in cellular c-Fos expression after treatment with EGF, an activator of the MAPK pathway. These results indicate that the elevation of c-Jun in breast cancer cells is controlled post-transcriptionally by a mechanism that is not dependent on the MAPK pathway.

**Figure 4:**
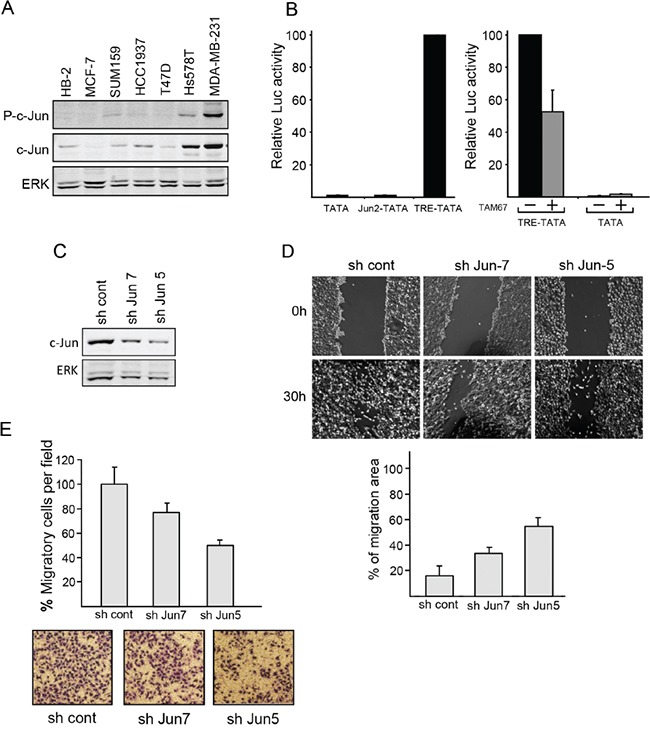
c-Jun contributes to the malignant properties of breast cancer cells **A.** Western blot analysis of phosphor c-Jun (P-c-Jun), c-Jun and ERK expression in breast cell lines, as indicated. **B.** MDA-MB-231 cells were transfected with the reporter constructs TATA, TRE-TATA or Jun2-TATA without (left panel) or with the dominant negative c-Jun construct TAM67 (+) or an empty vector (−) as control (right panel). In each experiment, luciferase activity obtained in the presence of the reporter construct TRE-TATA was given the arbitrary value of 100 and used to normalize all other results. The data shown are the means + SD of three separate experiments. **C.** MDA-MB-231 cells were stably transfected with c-Jun directed shRNA (sh Jun 5 or sh Jun 7) or with control shRNA (sh cont). The cellular levels of c-Jun and ERK were assayed by western blotting. **D.** Migration of the stably transfected cells versus control cells was measured by wound healing assay. Representative images of a wounded area are shown. The wound areas were measured after 30h using Ianugral++ software and presented as percentage of the wound area at the time point of scratching. Values are mean ± SD of four randomly chosen wound edges of four different scratches. **E.** Transwell migration of control and stably transfected cells was measured. Number of cells at the bottom of the transwell filters was counted after 4h and expressed as percentage of migrating cells per field relative to that of the control cells. Data presented as means ± SD of five different fields in two separate experiments.

The regulatory region of the c-Jun gene includes an AP-1 site through which c-Jun autoregulates its own transcription [1, 27]. The post transcriptional control of c-Jun levels in metastatic breast cancer cells could imply transcriptional inactivity of the protein. Transcription activity of c-Jun is enhanced by phosphorylation of serines 63 and 73 at the N-terminus of the protein [27]. Binding of anti-phospho-c-Jun antibodies confirmed phosphorylation of the accumulated protein (Figure 4A). Transcriptional activity of the accumulated c-Jun was also directly assayed by transfection of reporter constructs containing a minimal TATA box attached to five copies of the AP-1 sequence from the promoters of c-Jun (Jun2-TATA) or MMP1 (TRE-TATA). The results showed that expression of the TRE-TATA construct was 100-fold higher than that of the Jun2-TATA construct or a control construct lacking the AP-1 sequence (TATA), (Figure 4B). Cotransfection of TAM67, a dominant negative form of c-Jun, markedly reduced the expression of TRE-TATA (Figure 4B). This finding indicates that the c-Jun protein in metastatic breast cancer cells cannot activate its own promoter but is, nevertheless, transcriptionally active. To further evaluate the functional capabilities of c-Jun, we tested whether knockdown of c-Jun could affect the migration of metastatic breast cancer cells. Wound healing (Figure 4D) and transwell (Figure 4E) migration assays revealed that downregulation of c-Jun by stable transfection of c-Jun shRNA (shRNA7 or shRNA5) (Figure 4C) caused a considerable decline in migration capability, thereby demonstrating the contribution of c-Jun to the malignancy of the cells.

### miR-10b positively regulates the expression of c-Jun in metastatic breast cancer cells

**Figure 5:**
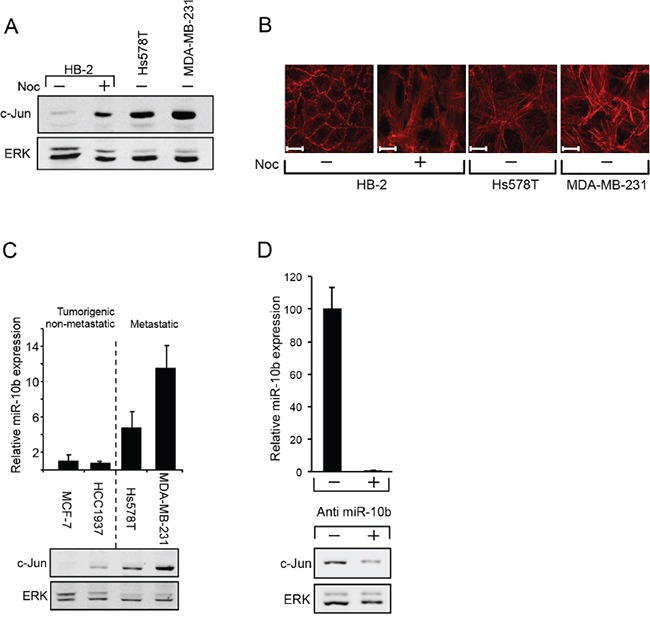
miR10b regulates the expression of c-Jun in breast cancer cells **A.** Protein samples from HB-2 cells, untreated (−) or treated (+) with nocodazole (Noc) for 18h were analyzed by western blotting using anti c-Jun or ERK Abs. **B.** Parallel cultures were fixed and stained with phalloidin to visualize the actin cytoskeleton (scale bar: 20μm). **C.** RNA samples from breast cancer cell lines were analyzed by quantitative RT-PCR using primers for miR-10b and U6 snRNA (upper panel). Parallel protein samples were analyzed by western blot using anti c-Jun and anti ERK antibodies (lower panel). **D.** MDA-MB-231 cells were transfected with antisense oligonucleotide against miR-10b (+) or with control oligonucleotide (−). RNA samples were analyzed by quantitative RT-PCR using primers for miR-10b and U6 snRNA (upper panel). Parallel protein samples were analyzed by western blot using anti c-Jun and anti ERK antibodies (lower panel). All experiments were repeated at least two times and representative immunoblots are shown.

The observation that the increase of c-Jun in metastatic breast cancer cells is post transcriptionally controlled without dependence on MAPK activity, raised the possibility that, as in the HaCaT cells, the increase is mediated by the miR-10b pathway. This could involve RhoC and NF1, which are known to alter cytoskeletal organization. To assess this possibility, we first examined whether restructuring of the cytoskeleton could increase c-Jun expression in non-tumorigenic breast epithelial cells (HB-2). Western blot analysis revealed that in common with other cell types [7, 9, 10], treatment with Nocodazole, a microtubule depolymerization agent, resulted in a marked rise in levels of c-Jun (Figure 5A). Histochemical analysis showed that this increase was accompanied by dramatic changes in cytoskeletal organization, as evidenced by the formation of extensive actin stress fibers. Similar changes were also seen in metastatic breast cancer cells, which also accumulate high levels of c-Jun (Figure 5B).

**Figure 6:**
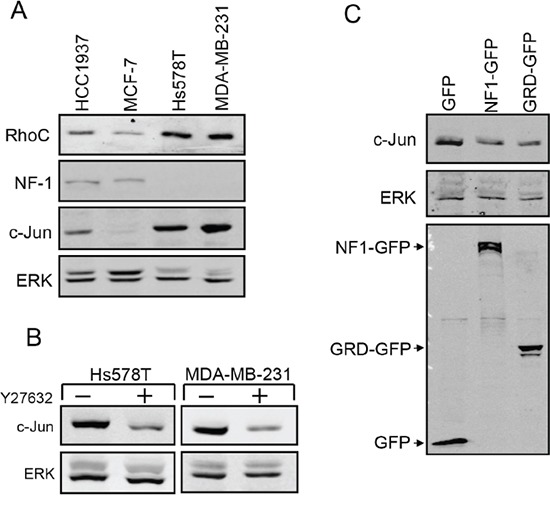
Analysis of RhoC and NF-1 in metastatic breast cancer cells **A.** Breast cancer cells at different stages of malignancy were analyzed by Western bolt using anti RhoC, NF1, c-Jun and ERK antibodies. **B.** Analysis of c-Jun and ERK expression in Hs578T and MDA-MB-231 cells treated (+) or untreated with Y27632. **C.** Hs578T cells were transfected with an expression vector for GFP, GFP tagged full length NF1 (NF1-GFP) or GFP tagged NF1 GRD domain (GRD-GFP). GFP expressing cells were isolated by FACS and analyzed by Western bolt using anti c-Jun, GFP and ERK antibodies. Experiments were repeated three times, and representative immunoblots are shown.

Analysis of miR-10b expression in breast cancer cells at different stages of malignancy revealed a direct correlation between c-Jun and miR-10b expression: Similarly to c-Jun, expression of miR-10b in metastatic breast cancer cells (Hs578T, MDA-MB-231) was considerably higher than that in non-metastatic cells (MCF-7, HCC1937) (Figure 5C). To determine whether silencing of miR-10b would affect the expression of c-Jun we used antisense oligonucleotides for miR-10b or oligonucleotides with a scrambled sequence as control. The oligonucleotides were transfected into metastatic breast cancer cells and silencing of miR-10b was assessed by quantitative RT-PCR (Figure 5D, upper panel). Analysis of c-Jun expression revealed that knockdown of miR-10b led to a decline in accumulation of c-Jun (Figure 5D, lower panel). A decline in expression of RhoC was also observed (Supplementary Figure S1). To examine whether here too, miR-10b affects c-Jun expression through RhoC and NF1, we measured their expression levels in metastatic and non-metastatic breast cancer cells. From Western blot analysis, the levels of RhoC were lower in non-metastatic cells than in metastatic cells, a trend opposite to NF1, where the relative levels were higher in the non-metastatic cells (Figure 6A). These changes appear to be directly related to the increased expression of c-Jun: Inhibition of ROCK, the main downstream effector of RhoC, by the addition of Y27632, markedly reduced the accumulation of c-Jun in the metastatic cells (Figure 6B). Similar results were obtained upon overexpression of NF1. This protein contains four domains, of which, the functional Ras GTPase-activating protein Related Domain (GRD), is responsible for most of the NF1 activity [28, 29]. Hs578T cells were transfected with GFP-tagged full length NF1 (NF1-GFP), GFP-tagged GRD domain (GRD-GFP) or with GFP alone, as control. GFP expressing cells were isolated using Florescence-Activated Cell Sorting (FACS) and analyzed by Western blot. The results showed that the expression of c-Jun was reduced by about 60% following overexpression of either the full length NF1 or the GRD domain (Figure 6C). In conclusion, these results suggest that miR-10b stimulates the expression of c-Jun in metastatic breast cancer cells through RhoC and NF1.

## DISCUSSION

In most tumors of epithelial origin the molecular program driving progression toward the invasive phase is the epithelial mesenchymal transition (EMT). This process is characterized by extensive cytoskeleton rearrangement and the functional loss of the cell-cell adhesion molecule E-cadherin. Previous studies have shown that this loss facilitates metastasis by allowing the separation of tumor cells from one another. Here we show that loss of E-cadherin also activates the miR-10b pathway, resulting in increased levels of c-Jun, a protooncogene that promotes the metastatic behavior of cancer cells.

Analysis of human breast cancer cells at different stages of malignancy confirmed that E-cadherin loss from metastatic cells is followed by a marked increase in c-Jun protein accumulation, with an accompanying promotion of the cell malignancy. Conversely, repression of c-Jun by shRNA reduces the migratory capacity of metastatic breast cancer cells. In line with these findings, there have been previous reports that overexpression of c-Jun in weakly invasive breast cancer cells increases invasiveness, migration, and hormone-independent tumor formation [30, 31]. Thus, c-Jun protein expression may serve as a diagnostic marker of breast cancer tumor progression and also represent a molecular target for therapeutic intervention.

In metastatic breast cancer cells RNA analysis showed that the expression of c-Jun is controlled post-transcriptionally since the elevated levels of c-Jun protein are not accompanied by an increase in c-Jun mRNA. Moreover, the accumulation of c-Jun protein was not dependent on MAPK, a major signaling pathway required for c-Jun transcription. Consistent with these findings, transfection experiments demonstrated that c-Jun is transcriptionally active against the AP1 sequence in the MMP1 promoter, even though there was no effect on the self-promoter. It is possible that the Jun/AP1 complexes formed in the cellular context of metastatic breast cancer cells can interact with the AP1 sequence of the MMP1 promoter, but not with that of c-Jun [3, 32]. Such conditions may prevent the transcription of c-Jun but facilitate transcription of the proteolytic enzymes that favor malignancy.

The situation in which there is an increase in c-Jun levels following the loss of E-cadherin is not unique to metastatic breast cancer cells. Previous studies have shown that both dispersion of cells and E-cadherin inhibition elevate c-Jun protein levels [7, 8], an increase that is translationally regulated by a mechanism that involves the cytoskeletal network [7]. There is ample evidence linking the cytoskeletal network, composed of microtubules and actin, to the cellular translation machinery. The cytoskeleton is (i) associated with translation machinery components, such as polysomes and factors that are involved in initiation and elongation of translation, (ii) mediates mRNA transport and (iii) regulates protein translation in an active manner (For review [33]). However, the nature of the signaling pathway responsible for the cytoskeleton-dependent upregulation of c-Jun translation has not yet been identified.

**Figure 7:**
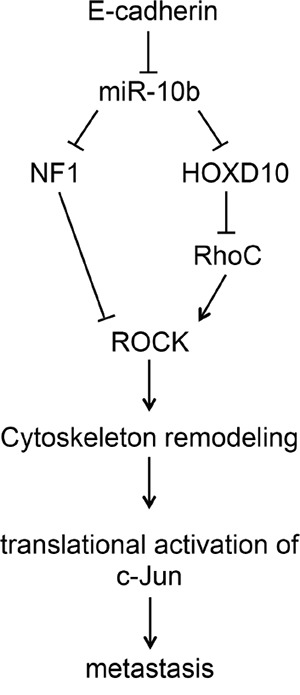
Upregulation of c-Jun by the miR10b pathway Loss of E-cadherin, which occurs during cancer progression, leads to an increase in miR-10b expression. miR-10b binds to a target sequence in the 3′UTRs of NF1 and HOXD10 and represses their expression. The decrease in HOXD10 causes an increase in the expression of RhoC, which activates ROCK, while the decrease in NF1 expression alleviates the inhibition of ROCK pathway. ROCK activation leads to changes in cytoskeleton organization which activates the translation of c-Jun and thereby promotes tumor cell invasion and metastasis.

In this study, we have demonstrated the critical role that the miR-10b signaling pathway plays in the activation of c-Jun. Loss of E-cadherin in HaCaT or metastatic breast cancer cells is accompanied by a marked induction of miR-10b. This increase might be mediated by the transcription factor Twist 1, which is reportedly upregulated upon loss of E-cadherin [34] and can activate the transcription of miR10b by binding to an E-box element in the regulatory region of the gene [13, 35]. Overexpression of miR-10b in HaCaT cells is sufficient to upregulate c-Jun, while knockdown of miR-10b in metastatic breast cancer cells leads to a decline in c-Jun expression. As illustrated schematically in Figure 7, downstream of miR-10b are two proteins, RhoC and NF1, which are inversely regulated by miR-10b. While miR-10b activates RhoC expression, probably through down regulation of the transcriptional repressor HOXD10 [13, 21], the expression of NF1 is repressed [20]. These two proteins are functionally associated with c-Jun regulation as shown by: (i) Overexpression of constitutively active RhoC in HaCaT cells increased c-Jun levels, while addition of Y27632 to inhibit ROCK, the main downstream effector, markedly decreased c-Jun accumulation. (ii) Overexpression of NF1 in metastatic breast cancer cells decreased c-Jun levels, while NF1 knockout in mouse embryonic fibroblasts elevated the accumulation of c-Jun considerably. Our results suggest the involvement of an undescribed regulatory pathway in which a specific microRNA, which alters cytoskeletal dynamics, activates the expression of the protooncogene c-Jun leading to tumor cell invasion and metastasis. Considering that miR10b is markedly elevated in tumor samples from patients with advanced-stage breast cancer [13, 36], pancreatic adenocarcinomas [37], and glioblastomas [38, 39], this c-Jun activating pathway might play an important part in determining the highly metastatic and invasive phenotypes of the cells.

## MATERIALS AND METHODS

### Reagents and plasmids

Nocodazole and peroxovanadate (VOOH) were purchased from Sigma. EGF was purchased from R&D systems. Y27632 was purchased from Tocris Bioscience and the MEK inhibitor, PD98059 was purchased from A.G. Scientific. The reporter constructs 5XcollTRETATA-Luc (TRE-TATA), 5Xjun2TRE-TATA-Luc (Jun2-TATA) and TATA-Luc (TATA) [32] were a gift from P. Angel (Heidelberg, Germany). The expression vector for dominant-negative c-Jun, pEGP-TAM67 [4], was kindly provided by R.F Hennigan, (New York, USA). Expression vector for miR-10b [40] was provided by Agami R. (The Netherlands Cancer Institute, Amsterdam). TK-Rnl (Promega), pCDNA3 (Invitrogen) and pEGFP-C3 (Clontech) are commercial vectors. The expression vector for NF1-GRD-GFP was provided by Kloog Y. (Tel Aviv University, Tel Aviv) and the full length NF1 (NF1-GFP) was a gift from McCormick F. (University of California, San Francisco). Constitutively active RhoC construct, RhoC G14V 3XHA-tagged, was purchased from Missouri S&T cDNA Resource Center. Anti-hsa-miR-10b (miScript miRNA Inhibitor) and control oligonucleotide were purchased from Qiagen. A panel of shRNA constructs for c-Jun and a control vector encoding non-effective 29-mer cassette, were purchased from OriGene Technologies, Inc. shRNA constructs with the strongest effect on c-Jun (c-Jun 5 and c-Jun 7) were used for further experiments.

### Cell cultures and fluorescence imaging

All cells were grown at 37°C in medium supplemented with 10% (vol/vol) FBS in a humidified atmosphere containing 5% CO_2_. MCF-7, SUM159, Hs578T, MDA-MB-231, HeLa, HaCaT and MEF cells were grown in DMEM. HB-2 cells were grown in DMEM supplemented with 10μg/ml insulin (Biological Industries), 5μg/ml Hydrocortisone (Sigma), and 1mM sodium pyruvate. T47D cells were grown in RPMI supplemented with 70μg/ml insulin. Cells were treated with drugs at the following end concentrations and period of time: Noc (0.5 μg/ml) for 18h, EGF (100 ng/ml) for 30 min, Y27632 (10μM) for 20h, peroxovanadate (0.1mM) for 15 min or PD98059 (30μM) for 4h. Actin cytoskeleton was visualized as previously described [7]. Confocal imaging was performed using Leica TCS STED confocal microscope (Leica microsystems).

### Isolation and quantification of RNA

Total RNA was isolated from cell cultures using the EZ-RNA reagent (Biological Industries), according to the manufacturer's instructions. For quantitative RT-PCR analysis, RNA was digested with RNase-free DNase (MBI Fermentas) to remove residual DNA and purified with LiCl. First-strand cDNA synthesis was performed using 1μg of total RNA and the Verso cDNA synthesis kit (Thermo Scientific) according to the manufacturer's instructions. Quantitative real-time PCR was performed using SYBR Green PCR Master Mix (Applied Biosystems) in total volume of 20μl containing: 10μl reaction mix, 400 nM of forward and reverse primers and 6μl of cDNA reaction for c-jun amplification, and 500 nM of forward and reverse primers and 2μl of cDNA for GAPDH amplification. The following oligonucleotide primers were used for the c-Jun transcript: 5′-GGATCAAGGCGGAGA GGAA-3′ (forward) 5′-GGGCGATTCTCTCCAG CTT-3′ (reverse) The following oligonucleotide primers were used for the GAPDH transcript: 5′-AGCCTCAAGATCATCAGCAATG-3′ (forward) 5′-GTCATGAGTCCTTCCACGATACC-3′ (reverse) Amplification and product detection were performed using ABI PRISM® 7700 Sequence Detection System (Applied Biosystems). Results were normalized to GAPDH expression and gene expression was calculated according to the ΔCt method. For microRNA analysis, cDNA synthesis was performed using 10 ng of total RNA and the TaqMan® MicroRNA Reverse Transcription Kit (Applied Biosystems) according to the manufacturer's instructions. Real-time PCR was performed as above, using TaqMan® Small RNA Assays (Applied Biosystems). U6 small nuclear RNA was used as an internal control.

### Global miRNA profiling

1 μg of total RNA was used to generate cDNA using the TaqMan Low-Density Arrays (TLDAs), which are quantitative real-time-polymerase chain reaction (RT–PCR) assays, based on specific stem–loop primers, each is complement to a mature miRNA (Life technologies). Many reactions are promoted in parallel by the primers mixture (multiplex PCR). First-strand Complementary DNA (cDNA) made with High Capacity cDNA kit, RNase-free water and TaqMan Universal PCR Master Mix (No AmpErase UNG; Life technologies) was then introduced into the loading ports on Human TLDA card A and B (671 miRNAs), centrifuged twice and sealed according to the manufacturer's instructions. PCR amplification was done on an ABI Prism 7900HT Sequence Detection System under the following conditions: 2 min at 50°C, 10 min at 95°C, 50 cycles of (30 s at 95°C and 1 min at 60°C). miRNA relative levels were calculated based on the comparative threshold cycle (Ct) method (see RQ calculation below). Reactions were run on an Applied Biosystems 7900HT Fast Real-Time PCR System. Normalization was achieved by reducing the Ct of each miRNA from the Ct of RNU44 ΔCt=(CtmiRNA – CtRNU44). For each miRNA, we reduced the normalized Ct in control cells from the normalized Ct in E-cad DN cells to create ΔΔCt values (CtE-cad DN cells - Ctcontrol cells). RQ number is calculated by 2 exponent the remainder from the last step (RQ=2−ΔΔCt).

### Protein preparation and western blot analysis

Cellular protein extracts were prepared as previously described [7]. Equal protein samples (40μg) were separated on 10% (15% for analysis of RhoC or 7.5% for analysis of NF1) SDS polyacrylamide gels and analyzed by Western blotting using Odyssey Blocking Buffer (LI-COR Biosciences) and the following antibodies: anti c-Jun and anti E-cadherin (Transduction Laboratories), anti RhoC (Abcam), anti ERK and anti phospho ERK (Sigma), anti-JNK, anti p38, anti phospho-c-Jun and anti c-Fos (Santa Cruz Biotechnology), anti phospho-JNK and anti phospho-p38 (Cell Signaling) anti GFP (Covance) and anti neurofibromin (Bethyl Laboratories). Anti-mouse or anti-rabbit IgG coupled to IRDye 800CW (LI-COR Biosciences) was used as secondary antibody, and protein bands were visualized by the Odyssey infrared imaging system (LI-COR Biosciences). Bend intensity was determined using the Odyssey software (LI-COR Biosciences).

### Transfections and luciferase assay

Cells (5×10^5^ per well) were seeded into six-well plates 24h before transfection. DNA (3μg) was transfected using jetPEI™ (Polyplus transfection), according to the protocols supplied. Protein extracts for immunoblotting were prepared 48h after transfection. Clones of HaCaT cells, stably transfected with miR-10b, RhoC or shRNA were selected in the presence of blacticidin (10μg/ml, A.G. Scientific), G418 (500μg/ml, A.G. Scientific) or puromycin (0.7 μg/ml, Sigma), respectively. MDA-MB-231 cells were transfected with 200pmol of anti-miR-10b or control oligonucleotide (Qiagen) using Lipofectamine 2000 reagent (Invitrogen). 24h after transfection, cells were harvested for western blot and quantitative RT-PCR assays. Firefly Luciferase (FL) reporter constructs were transfected together with TK-Rnl (RL), to control for transfection efficiency. FL and RL activities were assayed 48h after DNA transfection, as previously described [7]

### Florescence-activated cell sorting (FACS)

Hs578T cells (2.5×10^6^ per 10cm plate) were seeded 24h prior to transfection. EGFP expression vectors (8μg) were transfected using jetPEI™ (Polyplus transfection), according to the protocols supplied. 48h after transfection cell were harvested by trypsinization, washed twice with PBS and suspended in 0.5mM EDTA in PBS. EGFP expressing cells were separated using BD FACSAria IIu cell sorter (Becton Dickinson). Baseline fluorescence was obtained using untransfected cells.

### Migration assays

Migration was assessed by transwell and wound healing assays, as previously described [41]. For transwell assay, 1×10^5^ cells were plated in the upper chamber of 8μm pores transwells (Costar) in DMEM without serum. After 4h, the lower compartment was filled with medium containing 10% FBS used as a chemoattractant, and cells were allowed to migrate for 4h. Samples were fixed with ethanol and stained with Diff-Quik kit (Dade Behring). The upper chambers were wiped with cotton swabs to remove non-migrating cells, and cells on the lower surface were photographed using OLYMPUS IX70 microscope. Five fields were photographed for each chamber and cells were counted. Statistical analysis was performed using Student's t test. For wound healing assay, cells were seeded on six-well plates and grown to confluency. Medium was replaced with medium containing 0.1% FBS, monolayers were scratched using pipette tip and incubated for 30h. Images of the wounded area were collected at the indicated time points using OLYMPUS IX70 microscope and X4 objective. Wound area was measured using Ianugral++ software.

## SUPPLEMENTARY FIGURE


