# Supplementary material for: MicroRNA 10b promotes abnormal expression of the proto-oncogene c-Jun in metastatic breast cancer cells
Source: Oncotarget. 2016 Aug 2;7(37):59932–44. doi: 10.18632/oncotarget.11000 (PMC5312359; doi:10.18632/oncotarget.11000)
Supplement: Supplementary file 1 [file oncotarget-07-59932-s001.pdf]

MicroRNA 10b promotes abnormal expression of the proto-oncogene c-Jun in metastatic breast cancer cells

SUPPLEMENTARY FIGURE

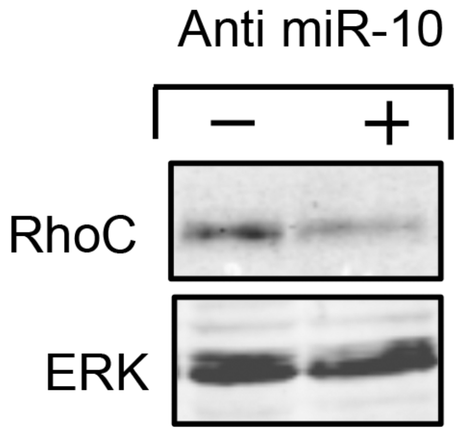

Supplementary Figure S1: Analysis of Rho C in metastatic breast cancer cells. MDA-MB-231 cells were transfected with antisense oligonucleotide against miR-10 (+) or with control oligonucleotide (-). Protein samples were analyzed by western blot using anti Rho C and anti ERK antibodies.
